# Practical Therapeutics, Considered Chiefly with Reference to Articles of the Materia Medica

**Published:** 1866-12

**Authors:** 


*Practical Therapeutics, considered chiefly with reference to Articles of the Materia Medica. By Edward John Waring, F. R. C. S., F. S.* S., *Surgeon in Her Majesty's Indian Army. From the Second London Edition. Lindsay and Blackiston, Philadelphia,* 1866.

The above work in one volume of 774 pages, with a full index of medicinal agents, and also of the diseases alluded to in connection with them, has been sent us by the publishers. Of its particular merits, we hope hereafter to be able to allude.

